# Biomimetic Effect of Saliva on Human Tooth Enamel: A Scanning Electron Microscopic Study

**DOI:** 10.1155/ijod/1664620

**Published:** 2025-01-04

**Authors:** Rozina Akter, Mohammad Ali Asgor Moral, Khalequzzaman Md, Bashar A. K. M.

**Affiliations:** ^1^Department of Conservative Dentistry and Endodontics, Bangabandhu Sheikh Mujib Medical University, Dhaka, Bangladesh; ^2^Department of Public Health and Informatics, Bangabandhu Sheikh Mujib Medical University, Dhaka, Bangladesh

**Keywords:** biomimesis, saliva, scanning electron microscopic study, tooth enamel

## Abstract

**Introduction:** Due to the presence of ion reservoir, saliva may facilitate enamel remineralization and neutralize pH of acidic beverage leads to prevent enamel demineralization. Saliva substitute/artificial saliva has been developed in subsequent years and may differ in physical properties, function, or pH level from 5.0 to 7.3.

**Objectives:** To evaluate the biomimetic effect of saliva (neutralization) on tooth enamel exposed to carbonated beverage (pH 2.44) and to observe therapeutic capability (remineralization) of artificial saliva over previously eroded (grade 3 and grade 5) enamel surface.

**Methods:** After scanning with electron microscope (SEM-EDX), nondemineralized crown samples (*n* = 40) were randomly grouped into two. Samples (50%) were flushed all around to carbonated beverage with collected natural saliva bathing simultaneously (experimental group, *n* = 20), and the rest flushed to beverage only without saliva bathing simultaneously (control group, *n* = 20). Flushing action was performed for 3 min by a customized digital automatic flusher for 30 times for each sample. Samples (*n* = 40) were further scanned under SEM-EDX to evaluate the demineralization grade and concentration of Ca, P, O, and C elements of crown samples to find out the neutralization effect of saliva. In the second phase, already demineralized crown samples (*n* = 30) were randomly treated with artificial saliva having two different pH (7 or 6.8, experimental groups) and distilled water (control group) for 15 min 3 times daily for 30 days. The remineralization score of experimental samples was graded, and therapeutic capability was established.

**Results:** Samples, when exposed to a carbonated beverage with saliva bathing simultaneously, showed low level of demineralization (mean 2.9 ± 0.3) than the control (without saliva) (mean 4.8 ± 0.3) (*p* = 0.01) which indicated neutralization (bioimimetic) effect of natural saliva. All (100%) of demineralized samples treated with both artificial saliva (pH 7 or pH 6.8) showed significant remineralization (*p* = 0.01), thus revealed biomimetic capacity. SEM-EDX analysis showed initial (before beverage exposure) concentrations of calcium, phosphorus, oxygen, and carbon elements of crown samples were 32.48%, 31.5%, 28.3%, and 5.5%, respectively. The calcium (Ca) (9.7%) and phosphorous (P) (18.5%) values were more decreased after beverage exposure without saliva bathing simultaneously compared to after beverage exposure with saliva bathing simultaneously. The concentration of oxygen (54.4%) and carbon (15.5%) were more increased after beverage exposure without saliva bathing simultaneously compared to after beverage exposure with saliva bathing simultaneously. Though the concentration of calcium (38.5%) of the crown sample was increased after treatment with artificial saliva (pH 7), but the phosphorus (18.5%) concentration of the crown sample was not increased.

**Conclusion:** Within the context of the present study, both natural and artificial saliva showed significant biomimetic effects with respect to neutralization and remineralization.

## 1. Introduction

When oral cavity is in contact with any acidic beverages, within a minute, salivary flow is stimulated and increases, and the bicarbonate ions in saliva react with the hydrogen ions released from acids to protect the enamel from demineralization, but the mimetic capacity of saliva can be overwhelmed by frequent or long-term exposure to acids [1, 2]. Saliva may play a dual role by (i) delivery of Ca^2+^and PO4^3−^ directly on enamel surface, which leads a super saturation that causes remineralization of tooth enamel, and (ii) gradual release of Ca^2+^ and PO4^3−^ ions leads a supersaturation that may cause remineralization of tooth enamel [3, 4].

The threshold pH value of a solution/beverage for enamel demineralization is less than 5.5 [4]. The threshold pH value is the value when solution (saliva or any liquid) or beverage is saturated with mineral particles of enamel. When the pH value of the beverage is below 5.5 causes demineralization of the enamel because the beverage is not saturated [5, 6]. Other hand, when the pH value of the solution/beverage is more than the threshold pH value of 5.5 leads to remineralization/biomimetic synthesis due to the solution is considered oversaturated [7].

Any acidic solution/beverage may dissolve the mineral components of enamel structure of tooth; hydrogen ions from solution/beverage reacts with carbonate and phosphate ions as the chemical equation shows below [8]:

  $$\begin{array}{c} \begin{array}{c} ↓↑\\ \left(10-x\right){\mathrm{Ca}}^{2+}+{x\mathrm{Na}}^{+}+\left(6-y\right)\left({\mathrm{HPO}}_{4}^{2-}\right)+y\left({\mathrm{HCO}}_{3}^{-}\right)+H_{2}O+{\mathrm{uF}}^{-}. \end{array} \end{array}$$

But, simultaneously, human saliva causes the calcium and phosphate ions to mobilize, consequently leads the pH value rises [9]. For the purpose of moisturizing and lubricating the oral tissues, human saliva is produced continuously [9, 10], and saliva is considered an ion reservoir solution and supersaturated with respect to tooth minerals may cause remineralization of tooth enamel. The buffering capacity of saliva also helps to neutralize low pH level of acidic beverage/solution, thus prevent enamel demineralization [10, 11]. The biomimetic synthesis/remineralization of enamel-like apatite structures is the regeneration of the demineralized enamel structure [12].

Several salivary parameters may be responsible for enamel remineralization or biomimesis; salivary pH level range 6.2–7.6. Salivary Ca^2+^ ions concentration varies from 1.03 to 3.6 mmol/L or 3.6 mmol/L = 3.6 × 10^−3^ mol/L = (3.6 x 10^−3^) × (6.022 × 10^23^) ions = 21.67 × 10^20^ ions. Salivary PO_4_^3−^ ionsconcentration is 4.5–6 mmol/L or 6 mmol/L = 6 × 10^−3^ mol/L = (6 × 10^−3^) × (6.022 × 10^23^) ions = 36.13 × 10^20^ ions [12, 13]. So, saliva is considered supersaturated with calcium and phosphate ions needed to prevent demineralization of the enamel surface of tooth [14]. Moreover, the salivary buffering capacity and salivary pH are determined by the hydrogen bicarbonate balance in saliva [15]. When the enamel of the teeth lose structure due to demineralization, salivary calcium, and phosphate ions may play to role in the neutralization (biomimesis) or constantly replace (remineralization) of any loss of tooth structure due to demineralization [16].

On the other hand, a diminished supply of saliva is associated with complications that increase suffering and lead to many oral diseases. Despite many therapeutic options, the use of saliva substitutes seems to be an effective solution. The substitutes available only for lab use for dental research vary in properties as well as in pH level [17]. Research in the field of development of artificial saliva has been focusing on groups of patients accompanied by a decrease in salivary secretion [18]. The development of such preparations only focuses on the function, such as extended antimicrobial or lubricating activity in patients with mucositis/xerostomia [18, 19].

The research field of development has been trying to reproduce the artificial saliva that fulfills the structural–functional relationships of individual salivary molecules. Although 99.5% of saliva is water, the molecules form a complex structure [20]. A four-level structural model of saliva consists of a liquid phase of the electrolyte solution, a scaffold-like continuous network structure, less water-soluble proteins, and other salivary molecules suspended in the network structure [20, 21].

In previous studies, the effectiveness of the composition of biologically active substances of artificial saliva such as Na, Cl, Zn, Fe, Mg, Cu, and organic compounds: indole and phenolic, triterpene saponins [22]. Comparison of the composition of artificial saliva in the discussed models showed in previous studies was very diverse, ranging from three components (NaHCO_3_, NaCl, and KCl) [23]. The pH of artificial saliva also varies from 5.0 to 7.3 [23, 24]. Applied pH depended on the purpose of the dental experiment. However, many research hypothesized the artificial saliva preparations may have the potential to mimic enamel structure physically, and it is expected to have a biomimetic capacity similar to normal human saliva [24]. In artificial saliva, (i) mucin, carboxymethyl cellulose, and glycerin are used to mimic natural saliva (viscosity), (ii) mineral content (calcium, phosphate, and fluorideions), (iii) preservatives such as methyl-or propylparaben), and (iv) palatability (flavorings mint, sorbitol, and xylitol) [25]. It is essential to evaluate that the ionic composition of saliva substitute can remineralize or not on an already demineralized enamel surface. Additionally, there was scarce of studies regarding the studies about qualitative direct visual analysis of enamel structural features under a scanning electron microscope (SEM) [25]. The objective of this study was to evaluate the changes in the enamel surface/prism morphology to determine demineralization patterns as well as the remineralization effect of artificial saliva by direct visual image analysis. Scanning with electron microscopic investigation (SEM-EDX analysis) was recommended as a fruitful evaluation technique for the direct qualitative analysis of the enamel structure [25, 26].

## 2. Materials and Methods

### 2.1. Phase I

**Figure 1:**
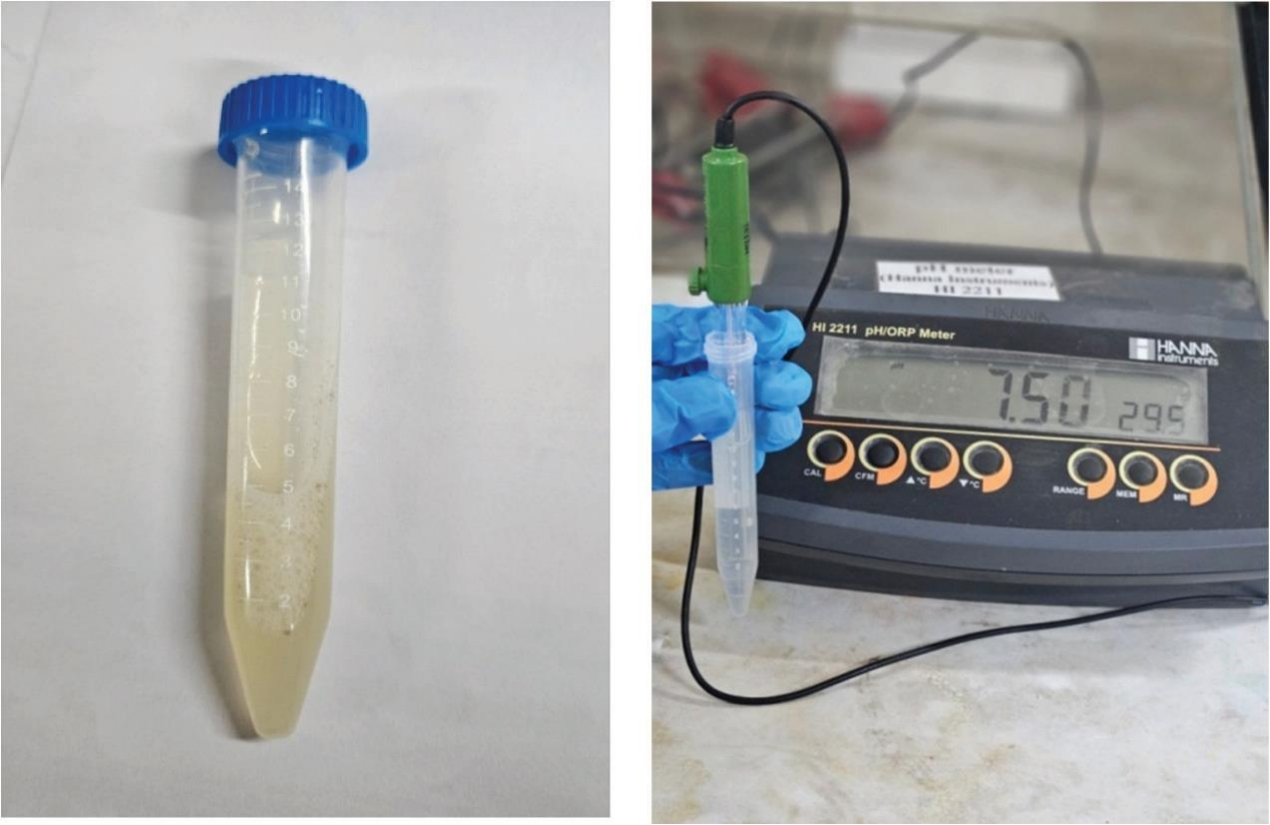
Collected natural saliva, pH of the natural saliva.

An in vitro was conducted to illustrate the demineralization level of enamel after exposure to carbonated beverage (pH 2.44) with and without natural saliva bathing simultaneously. The ingredients of carbonated beverage (CocaCola) are composed of carbonated water, sugar, acidity regulator-phosphoric acid (338), caramel (150 D), and caffeine. Natural saliva (pH 7.5) (Figure 1) was collected using the passive drool method from a healthy individual aged 41 years of old (female) following a brief rinsing of the mouth with water at noon time of the day and pH of the natural saliva was evaluated by pH meter (HANNA) at Department of Chemical Engineering, BUET, Dhaka.

Extracted human permanent premolar teeth were collected by purposive sampling technique and stored in distilled water at room temperature. Tooth samples were free of enamel erosion, dental caries, metallic restoration, fractured tooth, shape and structure anomalies, and external resorption. Tooth roots of samples were discarded with micromotor, and the lingual surface of crown sample was flatted for placing on the stab of the SEM machine.

**Figure 2:**
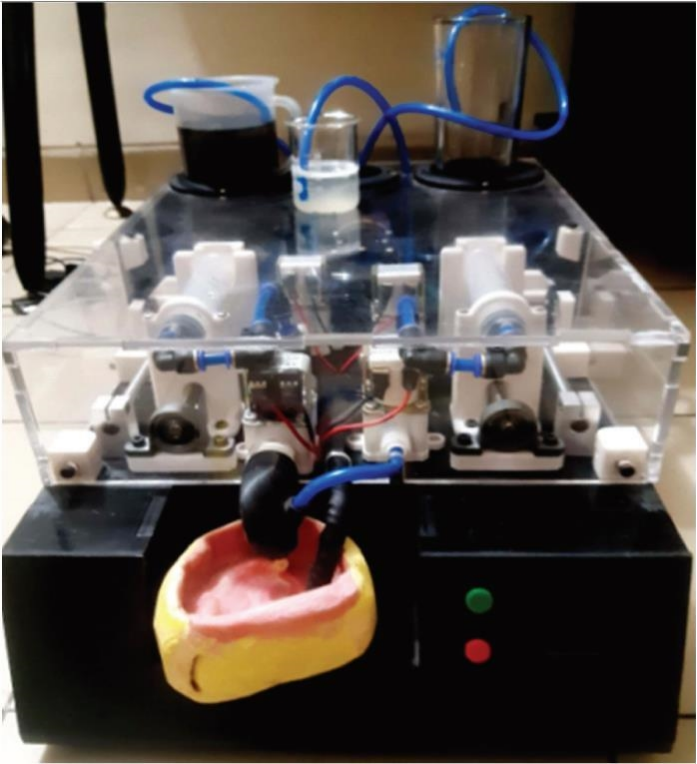
Customized digital automatic flusher used to beverage exposure with and without saliva simultaneously.

First, crown samples were dried in oven at 37°C for 7 days and mounted with gold sputtering. Buccal surface (middle) of the crown enamel was scanned under SEM at a magnification of 10,000 × to evaluate the existing morphological features of enamel surface. Total 40 nondemineralized samples were randomly grouped into two (*n* = 20). Samples (50%) were flushed all around to carbonated beverage with collected natural saliva bathing simultaneously (experimental group, *n* = 20), and the rest were flushed to beverage only without saliva bathing simultaneously (control group, *n* = 20). Flushing action was performed for 3 min by a customized digital automatic flusher (Figure 2), INVOLUTE Tech Limited, Dhaka, Bangladesh. The total amount of beverage for a single exposure/experimental trial was 250 mL, and the total amount of saliva for a single exposure/experimental trial was 16 mL.

**Figure 3:**
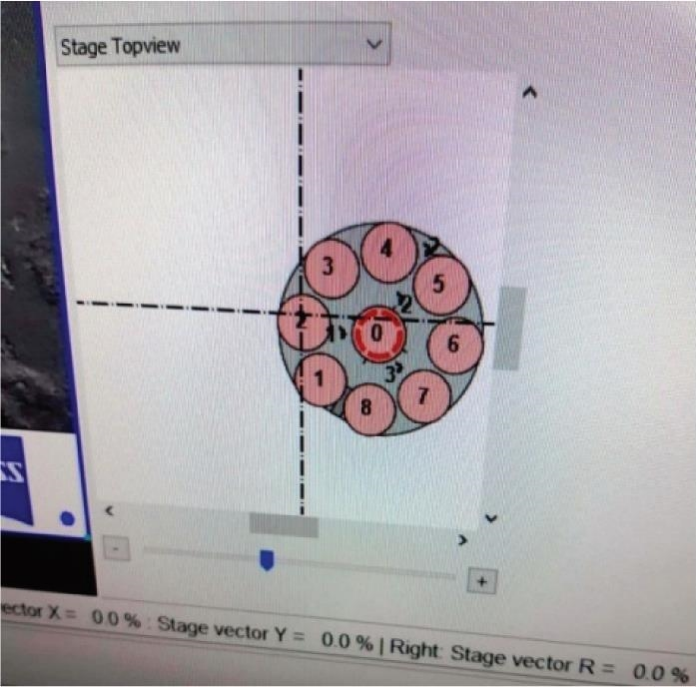
Pont out/fix the middle of the buccal surface of crown enamel with sample placing chamber of SEM machine. SEM, scanning electron microscope.

Samples (*n* = 40) were further scanned under SEM at the same magnification. The author scored the surface changes from the same area (point out/fix the middle of the buccal surface of crown enamel with sample placing chamber) (Figure 3) obtained before and after flushing the samples to evaluate the demineralization grade/score of the two groups following Galil and Wright's criteria [13] (Table 1) and compared within to find out the biomimetic effect of saliva. The representative digital photomicrographs (SEM image) were evaluated individually by two evaluators with the attributed evaluation scores.

### 2.2. Phase II

**Figure 4:**
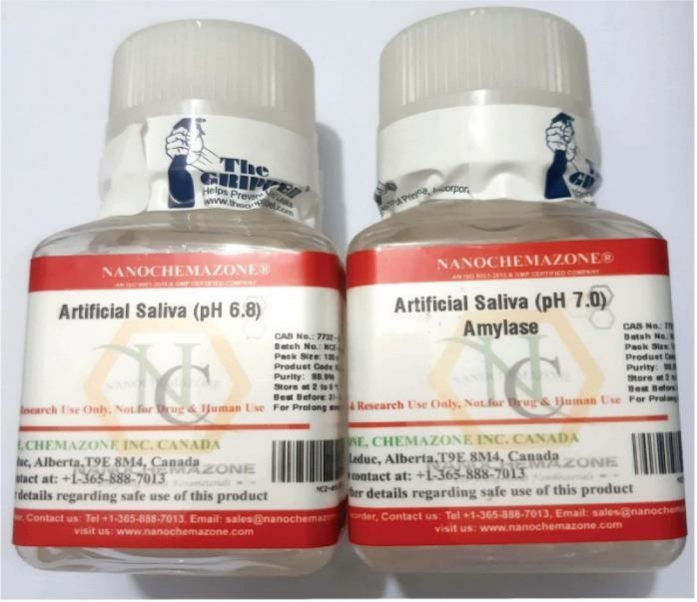
Artificial saliva.

Already demineralized crown samples (demineralization grade-3 and grade-5) (*n* = 30) were randomly treated with artificial saliva (Figure 4) having two different pH (7 or 6.8, experimental groups) and distilled water (control group). Samples were randomly attributed to three groups; group I: artificial saliva (pH 7) (*n* = 10), group II: artificial saliva (pH 6.8) (*n* = 10), and control group: distilled water (*n* = 10). After treatment with artificial saliva for 15 min 3 times daily for 30 days for each sample, the buccal surface of samples was further scanned under SEM at the same magnification. The representative digital photomicrographs (SEM image) were evaluated individually by two evaluators. Finally, the biomimesis/remineralization score of experimental samples was graded according to Rovery et al. [19] (Table 2) to establish the therapeutic capability of artificial saliva.

Furthermore, the mass normality (%) of calcium, phosphorus, carbon, and oxygen elements of the crown samples/enamel surface (10 µm) was evaluated with SEM-EDX analysis among initial crown samples (before beverage exposure) (*n* = 20), after beverage exposure with saliva bathing simultaneously (*n* = 10), after beverage exposure without saliva bathing simultaneously (*n* = 10), after treatment with artificial saliva (pH 7) (*n* = 10) and after treatment with artificial saliva (pH 6.8) (*n* = 10).

### 2.3. Statistical Analysis

All variables were analyzed by descriptive analysis and presented as frequency and percentage. The mean and standard deviation of the demineralization score were calculated with one-sample *T* test and shown in tables. To compare the remineralization or biomimetic score between experimental and control groups, cross-tabulation (Chi-square test) was done when *p* − value ≤ 0.05 considered as significant value.

## 3. Results

Samples, when exposed to a carbonated beverage with saliva bathing simultaneously, showed low level of demineralization (mean 2.9 ± 0.3) than the control (without saliva) (mean 4.8 ± 0.3) (*p* = 0.01) which indicated neutralization (bioimimetic) effect of natural saliva (Table 3).

All demineralized samples (100%) treated with distilled water showed no remineralization capacity (score-1), but 100% of demineralized samples treated with both artificial saliva (pH 7 or pH 6.8) showed significant remineralization (*p* = 0.01) thus revealed biomimetic capacity (Table 4).

After treatment with both artificial saliva (pH 7) and artificial saliva (pH 6.8), fully remineralization capacity (score-4) was revealed to 40% of samples, and 60% of samples showed moderate remineralization capacity (score-3) (Table 4).

SEM-EDX analysis showed initial (before beverage exposure) mean concentration of calcium, phosphorus, oxygen, and carbon elements of crown samples were 32.4 ± 0.72, 31.5 ± 0.79, 28.3 ± 0.86, and 5.5 ± 0.68, respectively. The calcium (Ca) (9.7 ± 1.0) and phosphorous (P) (18.5 ± 0.89) values were more decreased after beverage exposure without saliva bathing simultaneously compared to after beverage exposure with saliva bathing simultaneously. The mean concentration of oxygen (54.4 ± 0.95) and carbon (15.5 ± 0.99) were more increased after beverage exposure without saliva bathing simultaneously compared to after beverage exposure with saliva bathing simultaneously. Though the mean concentration of calcium (38.5 ± 0.98) of the crown sample was increased after treatment with artificial saliva (pH 7) but the phosphorus (18.5 ± 0.61) concentration of the crown sample was not increased (Table 5).

## 4. Discussion

**Figure 5:**
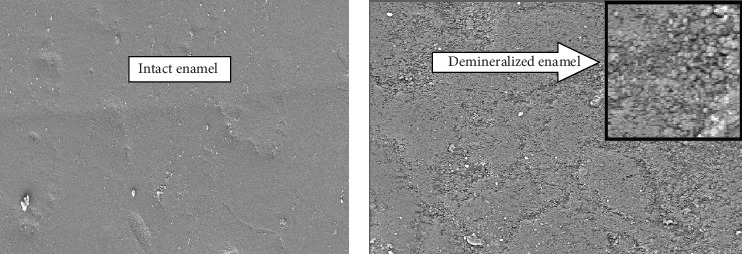
(a) Enamel structure before beverage exposure/intact enamel surface and (b) enamel structure after beverage exposure with saliva bating simultaneously and scale bar showing demineralized enamel surface.

**Figure 6:**
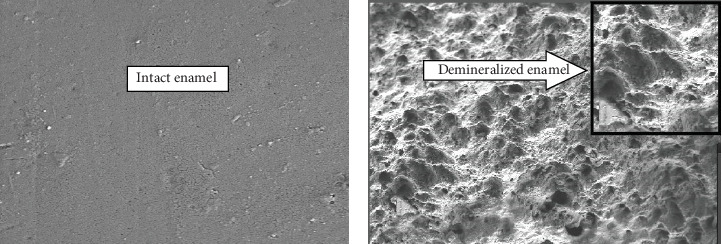
(a) Enamel structure before beverage exposure/intact enamel surface and (b) enamel structure after beverage exposure without saliva bating simultaneously and scale bar showing demineralized enamel surface.

In the present study majority of samples exposed to beverage with saliva simultaneously revealed the demineralization pattern showing superficial dissolution of the only enamel surface, not prism structure, giving “cobblestone” appearance (Grade-3) (Figure 5). But, when samples were exposed to beverage only without saliva bathing, the majority of samples revealed a demineralization pattern indicating preferential dissolution of the outer enamel surface as well as inner prismatic structure, giving random demineralization patterns both “cobblestone and honeycomb” appearance (Grade-5) (Figure 6).

The biomimetic (neutralization) effect of natural saliva, as clearly became evident in the present study finding, is supported by the study of Mulic et al. [1]. They observed erosion of teeth in young adults and found that those adults had got low salivary flow rates were affected by more erosion than those having normal salivary flow. Zimmer et al. [2] reported that men with erosion consumed twice carbonated beverages and held beverage in mouth 70% longer than men who had no erosion. Similarly, Toole et al. [3] found an association between incisal tooth erosion and the habit of holding beverages in mouth prior to swallowing among 6-year-old child. Both of these also favor the neutralization effect of saliva, as observed in the present study.

In previous studies, direct immersion of enamel sample in beverage was done in vitro setting [4, 5]. But, in this present study, a simulation of intraoral conditions could be achieved with the help of a customized digital automatic flusher was developed by INVOLUTE Tech Limited, Dhaka, Bangladesh. Here acidic beverage was flushed in the presence of saliva on the enamel surface to measure the demineralization level. Thus, an environment that can simulate the real-life conditions (inside a person's oral cavity/mouth) for the experiment was created to achieve the best suitable result. Therefore, all necessary conditions, like the simultaneous presence of natural saliva inside the mouth and periodic consumption of beverage at a certain volume (250 mL) in a specified time (3 min), are considered.

**Figure 7:**
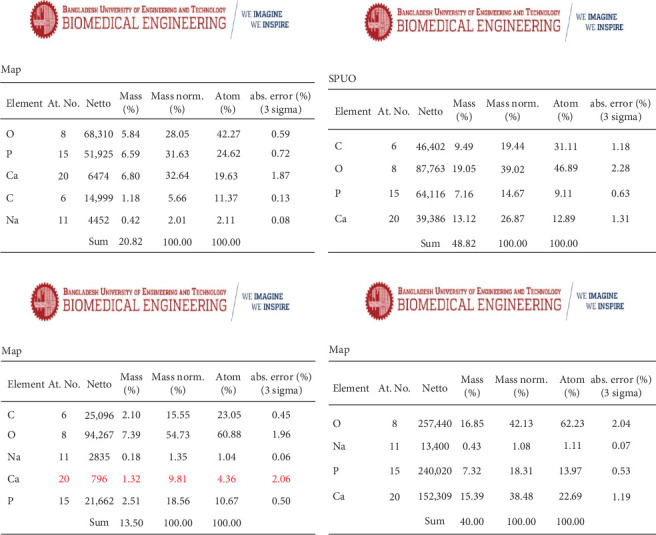
(a) Initial concentration (%) of Ca, P, O, C elements of the crown samples, (b) the concentration (%) of Ca, P, O, C elements of the crown samples after beverage exposure with saliva bathing simultaneously, (c) the concentration (%) of Ca, P, O, C elements of the crown samples after beverage exposure without saliva bathing simultaneously, and (d) the concentration (%) of Ca, P, O, C elements of the crown samples after treatment with artificial saliva.

SEM-EDX analysis showed that the calcium (Ca) and phosphorous (P) values were more decreased after beverage exposure without saliva bathing simultaneously compared to after beverage exposure with saliva bathing simultaneously (Figure 7). Moreover, the concentration of oxygen and carbon was more increased after beverage exposure without saliva bathing simultaneously compared to after beverage exposure with saliva bathing simultaneously. These results indicated that the beverage exposure to enamel surface has the potential to demineralize the enamel structure, and the natural saliva also has a neutralization (biomimetic) effect to prevent enamel demineralization. Additionally, the concentration of calcium (%) was increased after treatment with both forms of artificial saliva (pH 7 and pH 6.8), but the phosphorus concentration (%) was not increased, which indicated that artificial saliva has the capability to remineralize the enamel structure.

**Figure 8:**
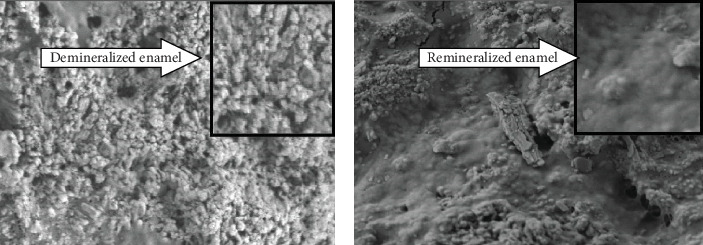
(a) Scale bar showing demineralized enamel structure before artificial saliva treatment and (b) scale bar showing remineralized enamel structure after artificial saliva treatment.

Demineralization and remineralization have a crucial effect on the hardness and strength of tooth enamel [5, 6]. It is considered that the strong and healthy tooth structure depends upon the ratio between demineralization and remineralization [7]. The present study showed a clear remineralization pattern (Figure 8) (appearance under SEM, remineralization grade-3 and grade-4) of previously eroded enamel samples with respect to immersion in artificial saliva having two different pH (pH 7 and pH 6.8). Whether demineralization or remineralization is proceeding at a time is determined by the balance between risk factors and protective factors [8, 9]. The product solubility (Ksp) is considered the effective concentration or activity of component ions. The concentration of component ions (Ksp) is expressed as mol/L [10]. Human enamel structure (hydroxypatite) has a static value of product solubility is 2.34 × 10^−59^. Usually, Ksp is a stable concentration for each component individually. The solution is saturated with elements constituting hydroxyapatite, and there is a balance between the concentration of ions and the concentration of products when Ip (concentration of ions) = Ksp. When the solution is not saturated, and demineralization takes place, if Ip less than Ksp. Remineralization takes place when the solution is oversaturated and Ip is more than Ksp [11, 12]. In the process of remineralization, calcium, and phosphate ions are supplied from an external source to the enamel structure to promote ion deposition into crystal voids in already demineralized enamel to produce mineral gain [13]. However, there are scarce studies about the theory that the saliva can play a role in remineralization by the release of Ca^2+^ and PO_4_^3−^ ions generating local supersaturation initiates the remineralization of enamel structure and recommendation of use of artificial saliva/therapeutic dose of artificial saliva [14, 15].

Hydroxyapatite (enamel structure) is semi-permeable due to the presence of a high percentage of mineral phases [16]. The interprismatic and intercrystalline coats (enamel pores) are formed as a consequence of the organic matrix formation, and those pores are capable to process of enamel diffusion [17, 18]. Like water, saliva is enable to pass with diluted ions and small molecules through the organic matrix between enamel crystals, leads a fluid flow depend on the morphology of the tooth enamel [18]. The structure of human enamel-Ca_10−*x*_ Na_*x*_ (PO_4_)_6−*y*_ (CO_3_)_*y*_ (OH)_2_ also contains about 2%–4% of carbonates and 1% of other chemical elements [19, 20]. Certain calcium ions may be substituted by other metal ions, such as sodium, magnesium, potassium, and phosphate ions are replaced by carbonate ones. Hydroxyapatite crystals increase its solubility due to the presence of carbonates and magnesium ions in it [21, 22].

In conclusion, within the context of the present study, both natural and artificial saliva showed significant biomimetic effects with respect to neutralization and remineralization.

### 4.1. Limitations of the Study

It was technically difficult to evaluate the rate of release of Ca^2+^ and PO_4_^3−^ ions, which generating the demineralization of the enamel structure. The effect of demineralization on enamel structure with direct exposure of beverage was not feasible to do in vivo setting. In addition, the biomimetic or remineralization effect of artificial saliva could not provide what extent does the application of artificial saliva remineralizes the already demineralized enamel structure with quantitative data.

### 4.2. Future Prospects of the Study

A prevention intervention program can be designed based on the demineralization/erosion effect of tested beverage on human enamel surface. It is expected that this study would provide the baseline data for the clinician about-

• Erosion or demineralization effect of carbonated beverage in different situations, such as the effect of only beverage exposure and the effect of beverage exposure with saliva simultaneously.
• The neutralization role of natural saliva is to protect the tooth structure from the demineralization by beverage consumption.
• The strategy focuses on increasing salivary secretion, particularly in compromised patients with xerostomia, to highlight the minimal intervention dentistry aims to preserve enamel tissue first and to emphasize the strategies available to aid in the prevention of erosion with history of beverage consumption by the oral motor therapy, pharmacological therapy to increase salivary flow.
• It is also hoped to provide the strategy of artificial saliva treatment to remineralize/replenish the enamel demineralization/erosion due to exposure of beverage, which will encourage to develop the therapeutic intervention or strategy for the management of patients with already eroded tooth.
• This study will also help our profession pride itself on an evidence-based approach to intervene in the general population regards the demineralization effect of carbonated beverage on tooth enamel in the highest standard possible and the strategy of therapeutic dose of artificial saliva.

## Figures and Tables

**Table 1:** Demineralization grade/score of enamel.

Demineralization score	Morphological features of enamel structure
Grade-1	No demineralization/change of structural morphological features of enamel surface

Grade-2	Distinct change of structural morphological features of enamel surface/loss of integrity of enamel surface

Grade-3	Etch pattern showing preferential dissolution of the enamel surface/prism peripheries giving a “cobblestone” appearance

Grade-4	Preferential dissolution of the enamel surface/prism cores resulting in a “honeycomb” appearance

Grade-5	A more random etch pattern corresponded to grade-3 and grade-4 together

Grade-6	Pitted enamel surface

**Table 2:** Remineralization score of enamel surface.

Enamel remineralization score	Morphological features of enamel surface
1	No remineralization/no change of demineralized enamel surface/the interprismatic and prismatic enamel structures totally appears/exposed

2	Any change of structural morphological features of enamel surface or less than 50% of demineralized enamel surface hidden by a homogeneous apatitic layer

3	Moderate remineralization/more than 50% of demineralized enamel surface hidden by a homogeneous apatitic layer

4	Fully remineralized/the interprismatic and prismatic enamel structures appear totally hidden/completely hidden demineralized enamel surface by a homogeneous apatitic layer

**Table 3:** Distribution of initial score of enamel surface, demineralization score after beverage exposure with and without saliva bathing simultaneously, mean and *p* (*n* = 40).

Sample no.	Initial enamel surface score (*n* = 20)	Demineralization score (DS) after beverage exposure with saliva (*n* = 20)	Mean of DS	Sample no.	Initial enamel surface score (*n* = 20)	Demineralization score (DS) after beverage exposure without saliva (*n* = 20)	Mean of DS	*p*
1	1	3	2.90 (± 0.30)	1	1	5	4.85 (± 0.36)	0.001
2	1	3	—	2	1	5	—	—
3	1	3	—	3	1	5	—	—
4	1	3	—	4	1	5	—	—
5	1	3	—	5	1	5	—	—
6	1	3	—	6	1	5	—	—
7	1	3	—	7	1	4	—	—
8	1	3	—	8	1	5	—	—
9	1	2	—	9	1	5	—	—
10	1	3	—	10	1	5	—	—
11	1	3	—	11	1	5	—	—
12	1	3	—	12	1	5	—	—
13	1	3	—	13	1	5	—	—
14	1	3	—	14	1	4	—	—
15	1	3	—	15	1	5	—	—
16	1	3	—	16	1	5	—	—
17	1	3	—	17	1	5	—	—
18	1	2	—	18	1	5	—	—
19	1	3	—	19	1	4	—	—
20	1	3	—	20	1	5	—	—

*⁣* ^*∗*^ *p* < 0.05 was considered statistically significant.

**Table 4:** Distribution of demineralized score of enamel samples and biomimetic synthesis/remineralization score of enamel surface after artificial saliva (AS) treatment (*n* = 30).

Study groups	Already demineralized enamel score	Remineralization score after artificial saliva and water treatment	*p*
Artificial saliva (pH = 7) (*n* = 10)	3	4	0.001
	3	4	—
	3	4	—
	3	4	—
	3	4	—
	5	3	—
	5	3	—
	3	3	—
	5	4	—
	3	4	—

Artificial saliva (pH = 6.8) (*n* = 10)	5	4	—
	5	3	—
	3	3	—
	3	3	—
	5	3	—
	5	3	—
	5	3	—
	5	3	—
	5	3	—
	3	3	—

Distilled water (pH = 7) (*n* = 10)	3	1	—
	3	1	—
	3	1	—
	3	1	—
	3	1	—
	3	1	—
	3	1	—
	5	1	—
	5	1	—
	5	1	—

*⁣* ^*∗*^ *p* < 0.05 was considered statistically significant.

**Table 5:** Distribution of mass normality (%) of calcium, phosphorus, carbon, and oxygen elements of the crown samples (enamel surface) (10 µm) with SEM-EDX anlysis.

Groups	Ca (calcium)	P (phosphorus)	O (oxygen)	C (carbon)
Initial samples (before beverage exposure) (*n* = 20)	32.48 ± 0.72	31.53 ± 0.79	28.31 ± 0.86	5.56 ± 0.68
After beverage exposure with saliva bathing simultaneously (*n* = 10)	27.63 ± 0.74	26.44 ± 1.2	33.35 ± 1.6	7.63 ± 0.79
After beverage exposure without saliva bathing simultaneously (*n* = 10)	9.74 ± 1.0	18.50 ± 0.89	54.45 ± 0.95	15.56 ± 0.99
After treatment with artificial saliva (pH 7) (*n* = 10)	38.53 ± 0.98	18.53 ± 0.61	42.38 ± 1.0	19.53 ± 0.88
After treatment with artificial saliva (pH 6.8) (*n* = 10)	37.24 ± 0.97	18.50 ± 0.85	41.50 ± 0.99	20.43 ± 1.0

Abbreviation: SEM-EDX, scanning with electron microscope.

## Data Availability

The data underlying this article will be shared on reasonable request to the corresponding author.
